# Exploring the effectiveness of demand-side retail pharmaceutical expenditure reforms: Cross-country evidence from weighted-average least squares estimation

**DOI:** 10.1007/s10754-022-09337-6

**Published:** 2022-09-21

**Authors:** Michael Berger, Markus Pock, Miriam Reiss, Gerald Röhrling, Thomas Czypionka

**Affiliations:** 1 Department of Health Economics Center for Public Health, Medical University of Vienna, Vienna, Austria; 2 Institute for Advanced Studies, Josefstädterstraße 39, Vienna, Austria; 3 London School of Economics and Political Science, Houghton Street, London, WC2A 2AE UK

**Keywords:** Public pharmaceutical expenditure, Health expenditure, Pharmaceutical policy, Panel data models, Weighted-average least squares, I18

## Abstract

Increasing expenditures on retail pharmaceuticals bring a critical challenge to the financial stability of healthcare systems worldwide. Policy makers have reacted by introducing a range of measures to control the growth of public pharmaceutical expenditure (PPE). Using panel data on European and non-European OECD member countries from 1990 to 2015, we evaluate the effectiveness of six types of demand-side expenditure control measures including physician-level behaviour measures, system-level price-control measures and substitution measures, alongside a proxy for cost-sharing and add a new dimension to the existing empirical evidence hitherto based on national-level and meta-studies. We use the weighted-average least squares regression framework adapted for estimation with panel-corrected standard errors. Our empirical analysis suggests that direct patient cost-sharing and some—but not all—demand-side measures successfully dampened PPE growth in the past. Cost-sharing schemes stand out as a powerful mechanism to curb PPE growth, but bear a high risk of adverse effects. Other demand-side measures are more limited in effect, though may be more equitable. Due to limitations inherent in the study approach and the data, the results are only explorative.

## Introduction

Past decades have seen steady increases in expenditure related to health, which has become a major issue in healthcare systems for many countries in terms of financial stability and sustainability. Expenditure on retail pharmaceuticals is a substantial component of total expenditure on healthcare and is easily traceable, making it a prime candidate for policy action. In 2015, this share was 16.2% on average across the 34 OECD member countries (excluding Chile, New Zealand and Turkey), but variation across countries is high, ranging from 6.8% in Denmark to 29.2% in Hungary, with a 37% coefficient of variation. With novel high-cost pharmaceuticals entering the market (e.g. in cancer care) and new therapies gradually shifting treatment out of the hospital sector (e.g. therapies for Hepatitis C), the pressure on policy makers to align healthcare and (retail) pharmaceutical expenditures with revenues will further increase.

**Fig. 1:**
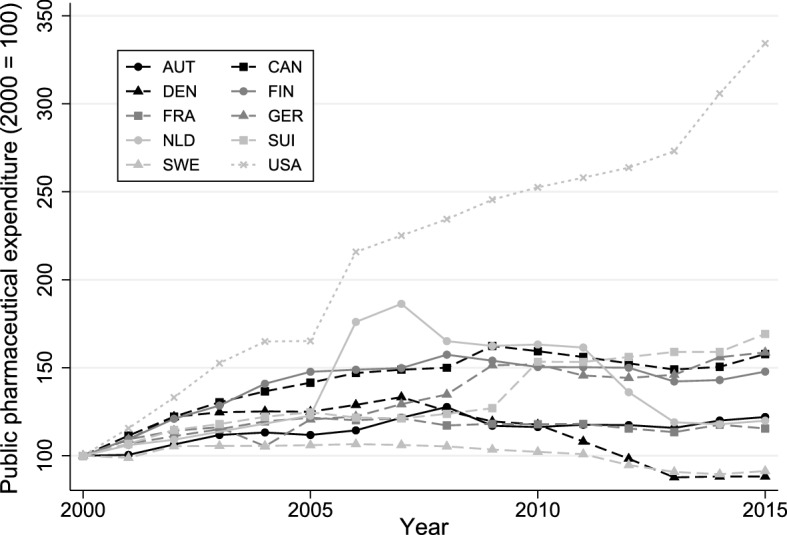
Real PPE growth in selected countries (national currency, constant prices). OECD Health Statistics (2017)

Recent trends in pharmaceutical spending in OECD countries are neither homogeneous over time nor across countries. From 2003 to 2015, public pharmaceutical expenditure (PPE) in OECD member countries (excluding Belgium, Chile, Israel, Latvia, Mexico, New Zealand, Turkey and the United Kingdom) increased by 1.1% on average in real terms per year. Splitting the sample in two six-year pre- and post-crisis periods highlights the varying dynamics. While real PPE growth rates were substantial prior to the crisis (3.6% yearly average), they turned negative on average in the post-crisis period. Figure 1 shows the development of real PPE of selected countries from 2000 to 2015. In terms of controlling costs, some countries performed better than others (with PPE declining in Denmark and Sweden, in contrast to steep growth by 234% in the US). In many countries, PPE growth rates still pressure public budgets. Belloni et al. (2016) suggest that future growth of pharmaceutical spending is likely to pick up pace again due to changes in pharmaceutical markets and increased availability of high-cost pharmaceuticals. Against this background, the question of appropriate expenditure control measures beyond direct price controls is again gaining importance.

The objective of this article is to evaluate the options available to policy makers to safeguard fiscal sustainability of healthcare systems across countries. We add to the existing literature by assessing the effectiveness of expenditure control measures for retail PPE in a cross-country panel setting using a core sample of 10 European OECD member countries (Austria, Belgium, Denmark, Finland, France, Germany, the Netherlands, Sweden, Switzerland, and the United Kingdom). We further use an extended sample of 12 countries (the core sample plus two additional non-European OECD member countries, Canada and the United States) to assess the robustness of the results. Over the last decades, governments have implemented a range of expenditure control measures on pharmaceuticals [for an overview see Vogler (2012)]. Some mainly target drug prices, others quantity and prescription behaviour. An overview of the relevant literature suggests that the evaluations of these control mechanisms for pharmaceutical expenditure have either been done on a national level (Bastida and Mossialos, 2000; Moreno-Torres et al., 2011; Barros and Nunes, 2010; Andersson et al., 2006, 2007; Lee et al., 2012; Fischer et al., 2018) or by meta-studies (Ghislandi et al., 2005; Galizzi et al., 2011; Tele & Groot, 2009; Acosta et al., 2014; Rashidian et al., 2015). Panel studies concerned with rising pharmaceutical expenditure mainly focus on income elasticity without considering policy variables (Okunade & Suraratdecha, 2006; Clemente et al., 2008). By pooling data from multiple countries, statistical inference gains power and findings stand independent of national context. It is imperative to identify and quantify the determinants of PPE growth to properly assess the effectiveness of expenditure control measures. At the same time, the impact of various expenditure control measures that have already been enacted must be evaluated. To the best of our knowledge, this is the first systematic empirical comparison of the effectiveness of pharmaceutical expenditure control measures in a cross-country setting. However, a limitation of this type of study is that the overlapping of several policies within countries at a given time does not allow to follow a clear identification strategy and our results are therefore first and foremost descriptive and hypothesis generating in nature and should not be read as causal. As we are concerned with the financial sustainability of public budgets, we focus our analysis on the part of expenditure borne by public entities. The analysis is further limited to policy reforms targeting retail pharmaceutical spending, i.e. pharmaceutical spending occurring outside hospitals, as the comparable System of Health Accounts methodology accounts for final consumption only. For a complete picture of PPE, pharmaceutical expenditure of the hospital sector would be desirable. However, we justify separate analysis by noting that pharmaceutical expenditure in hospitals need not have the same dynamics as retail pharmaceutical expenditure for several reasons. For instance, the majority of innovative, high-cost pharmaceutical interventions like cancer therapy takes place in a hospital setting. Pharmaceutical expenditure in hospitals may thus depend much more strongly on technological progress than retail pharmaceuticals. Country-specific healthcare regulations further exacerbate the different dynamics. In Austria and Germany, for example, the budget for hospital pharmaceuticals is a lump-sum part of the public hospital funding for diagnosis related treatment and therefore does not respond to direct price-damping measures of the public health funds for retail pharmaceuticals.

### Types of demand-side expenditure control measures

**Fig. 2:**
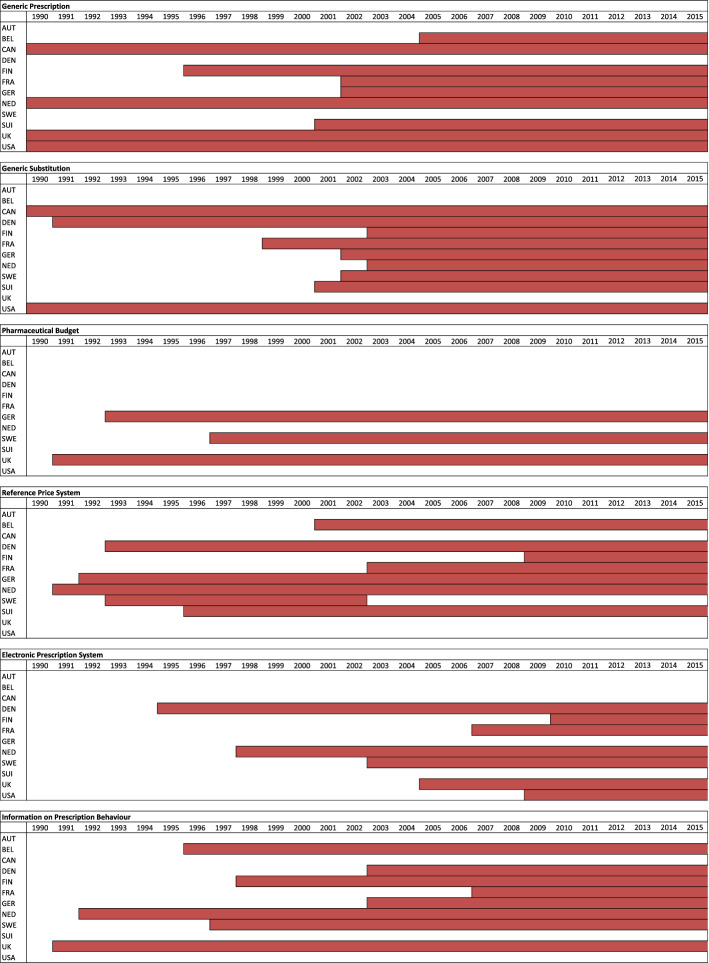
Timeline of the six principal categories of expenditure control measures per country in the data set

Following the Pharmaceutical Pricing and Reimbursement Information (PPRI) reports by the WHOCC (World Health Organisation Collaborating Centre for Pharmaceutical Pricing and Reimbursement Policies), the most common measures can be divided into six principal categories presented in Table 1. A graphical representation of the six principal categories as well as their timeline in the country sample of this analysis is provided in the appendix (Fig. 2). Although the practical implementation of the different reform types can differ, it is necessary to group them for cross-country analysis. Additionally, to allow for comparability across countries, only policies that were in place at the national level are considered in this analysis, thereby excluding (a) pilot projects (e.g. pilot projects for e-prescription in Switzerland), (b) policies only in effect at certain healthcare payers (e.g. the pharmaceutical budgets that some Austrian SHI funds implemented, but which were not coordinated on a national scale), and (c) policies only affecting specific groups of pharmaceuticals (e.g. generic substitution scheme in Belgium that is restricted to international non-propriety name (INN)-prescriptions, antibiotics and antimycotics, as well as pharmaceuticals with a reimbursement ceiling).

Measures that aim at the type and price of pharmaceuticals enjoy widespread use across OECD member countries. Substitution measures include (i) generic prescription, where physicians do not prescribe specific brands of drugs, but rather prescribe the drug’s generic name. The choice of drug is then left to the dispensing pharmacists. In contrast, with (ii) (compulsory) generic substitution, pharmacists are obliged to substitute a physician’s prescription of a brand drug with a generic drug, if available. Implementation of system-wide price-control measures such as (iii) reference price systems is also fairly common in OECD member countries. Reference price system in the context of our analysis is used in the meaning that pharmaceuticals are grouped according to their use, and one product’s price is set as a reference price. This procedure is often referred to as internal reference pricing. The public health system will then only cover that price. If a more expensive (brand) product is requested, patients have to pay the difference out-of-pocket. Internal reference pricing cannot be applied to (often highly-priced) innovative drugs that have only recently been introduced onto the market and for which no reference is available, potentially making the system less effective (Giuliania et al., 1998).^1^ In contrast, under external reference pricing, the price of a drug in one or several countries is used to derive a reference price. As such, external reference pricing is a typical pricing policy applied to innovative pharmaceuticals and is in effect in all countries included in the country sample for publicly reimbursed pharmaceuticals, though there is some indication that pharmaceutical producers have in the past tried to outmanoeuvre these mechanisms by systematically delaying dossier submissions in Belgium to avoid the lower Belgian prices to affect prices in other countries (Toumi et al., 2014). Moreover, the exact implementation of external reference pricing varies in practice. Several countries use external reference pricing only as a supplementary or initial pricing policy and Denmark restricts its use to the hospital sector altogether (Rémuzat et al., 2015). To account for this variation and the limitation of the dataset not covering hospital medication, the reference price system policy variable is limited to the form of internal reference pricing described above, which is applied in a more comparable fashion across countries. A similar problem exists for profit margin controls and Health Technology Assessment (HTA) for which an aggregation into policy variables is not possible, as the strategies pursued by countries are too different. Profit control is either set for wholesale or retail, or both, with the size of a fixed mark-up differing strongly between countries [see e.g. Lee et al. (2021)], and in countries with established HTA institutes, HTA may play a crucial role in the use of cost-intensive pharmaceuticals, even if there are no legal requirements to consider the results of HTA.

**Table 1:** The six principal categories of expenditure control measures

	Expenditure control measure category	Country (first year measure active in dataset)
(i)	Generic prescription	BEL (2005), CAN (1990), FIN (1996), FRA (2002), GER (2002), NLD (1990), SUI (2001), GBR (1990), USA (1990)
(ii)	Generic substitution	CAN (1990), DEN (1991), FIN (2003), FRA (1999), GER (2002), NLD (2003), SWE (2002), SUI (2001), USA (1990)
(iii)	Reference price systems	BEL (2001), DEN (1993), FIN (2009), FRA (2003), GER (1992), NLD (1991), SWE (1993-2002)$^{*}$, SUI (1996)
(iv)	Pharmaceutical budgets for physicians	GER (1993), SWE (1997), GBR (1991)
(v)	Electronic prescription	DEN (1995), FIN (2010), FRA (2007), NLD (1998), SWE (2003), GBR (2005), USA (2009)
(vi)	Information on prescription behaviour	BEL (1996), DEN (2003), FIN (1998), FRA (2007), GER (2003), NLD (1992), SWE (1997), GBR (1991)

*BEL* Belgium, *CAN* Canada, *FIN* Finland, *FRA* France, *GBR* United Kingdom of Great Britain and Northern Ireland, *GER* Germany, *NLD* The Netherlands, *SWE* Sweden, *SUI* Switzerland, *USA* United States of America

*Reference price system only in effect until 2002

Other methods tackle the problem directly at the physician level. Among these physician-level behaviour measures (iv) pharmaceutical budgets grant physicians a certain budget for the prescription of pharmaceuticals. If applied thoroughly, pharmaceutical budgets could potentially be a very effective cost control measure, but soft budget constraints (Kornai, 1986) can undermine the effects. Hard pharmaceutical budgets are, however, difficult to implement in practice. As exceeding a pharmaceutical budget may be medically justified in some instances, resistance by both physician and patients against hard budgets is likely. (v) Electronic prescription systems support physicians with finding the most economically sensible options among a choice of appropriate drugs. Another approach is to provide physicians with (vi) feedback on their prescription behaviour. By informing physicians about the cost structure and how their prescription behaviour compares to the national or regional average, management of costs and prescription behaviour is incentivised. This is an important measure in conjunction with pharmaceutical budgets. For policy bundles, it may also be possible to accrue an impact beyond that of simple additive effects when there are synergies between expenditure control measures. However, owing to the small sample size available and the limited amount of actually observed policy combinations, a detailed analysis is beyond the scope of our present study.

## Data

We use data from the OECD System of Health Accounts (SHA) dataset (OECD, Eurostat, WHO, 2017) in this analysis—with the exception of time series on the prevalence of overweight in adults, which is taken from the World Bank database (The World Bank, 2021). OECD data have the advantage that they are largely comparable across countries due to harmonized calculation methods, but which comes at the expense of a somewhat limited set of variables to choose from. In total, 12 OECD member countries were studied, for which data were sufficiently complete. The core dataset includes ten European countries (Austria, Belgium, Denmark, Finland, France, Germany, the Netherlands, Sweden, Switzerland, and the United Kingdom) while two non-European countries (Canada and the United States) were used to ascertain the robustness of the results in an extended dataset. Data were extracted from the OECD Health Statistics database for the period 1990–2015. As the dataset contained structural breaks and missing values, we performed uniform data cleansing to ensure sufficiently long and complete time series. Missing data points at the beginning or the end of time series were extrapolated with yearly average growth rates of the previous or subsequent periods, respectively. Missing values within time series without a structural break were linearly interpolated. Real structural breaks, for example, caused by a change in the method of calculating health expenditure, were smoothed by replacing the growth rate in the year of the break by the average growth rate of the two years preceding and succeeding the break.

As the dependent variable, we use log-differenced PPE per capita in national currency units at GDP prices of 2005. Independent variables cover a range of metric variables that are expected to influence the growth of pharmaceutical expenditure: on the demand side, log-differenced GDP per capita at 2005 prices is taken as a proxy for available income. Life expectancy at birth is used to account for differences in health expenditure owing to the demographic structure. There is some debate in the literature that healthcare expenditure is in principle determined by proximity to death rather than age [see e.g. Zweifel et al. (1999), Howdon & Rice (2018)]. However, such information cannot be extracted from macro time series, such as SHA data. Private expenditure on pharmaceuticals (through voluntary schemes or household out-of-pocket payments) as a share of total health expenditure is used as a proxy for private cost-sharing. Although information on private cost-sharing is provided in the SHA framework, this variable could not be used as too many data points were missing. On the supply side, the density of practicing generalist medical practitioners, specialists, physicians in total and pharmacists, and curative (acute) care beds per 1000 inhabitants were taken as resource variables to control for a potential connection between healthcare consumption and the density of healthcare provision through supplier-induced demand [e.g. Léonard et al. (2009)], as well as the setting in which healthcare is provided (e.g. the relative importance of specialised physicians in the outpatient sector vis-à-vis primary care physicians, or the extent of inpatient care), both of which may conceivably influence quantity and types of retail pharmaceutical prescriptions. The density of magnetic resonance imaging (MRI) units per 1 million inhabitants is used as a proxy for technological progress. For Belgium, Germany and Switzerland, for which data on the total density of MRI units were not available, we use MRI units in hospitals per 1 million inhabitants instead. As we are not interested in the density of MRI units as such, but only as a proxy for medical technological progress, mixing two time series does not limit our analysis. We control for epidemiological characteristics of the population with the percentage of overweight adults in the population to proxy the chronic disease burden (Kearns et al., 2014), and with the standardized death rate for malignant neoplasms per 100,000 inhabitants, which serves also as a proxy for access to cost-intensive treatments, as is the case with cancer (Kantarjian et al., 2014).

As differences in the financing structure of health systems may entail differences in expenditure on health and pharmaceuticals (e.g. through differences in bargaining position of payers vis-à-vis manufacturers, etc.), we further distinguish between countries whose healthcare system is primarily financed through social health insurance (SHI) contributions and those with a primarily tax-funded or otherwise funded healthcare system. For this purpose, we introduce a dummy variable in our regression model that takes the value 1 for SHI countries and 0 otherwise. Moreover, we proxy the role and importance of HTA in a country by a dummy variable that is 1 in case HTA is legislatively required in the process for reimbursement decisions.^2^ Note that due to the limitations in the accuracy of this variable definition—HTA may still impact reimbursement procedures, even if not explicitly legally binding—the estimated coefficient cannot be interpreted as a policy variable as such, but only as a control variable for a country-characteristic.

Finally, six dummy variables were constructed to assess the impact of the different expenditure control measures (see Table 1). Each measure takes the value 1 in each year the measure was in action and 0 otherwise. The summary statistics of the variables for both samples prior to the sequential correction of the error term structure are provided in Table 2.

**Table 2:** Summary statistics of the variables prior to the error term correction procedure

Summary statistics					
Variable	Observations	Mean	SD	Min	Max
*Core Sample*					
Public pharmaceutical expenditure (log-diff)	250	0.033	0.073	−0.322	0.433
Private pharmaceutical expenditure (log-diff)	250	−0.013	0.136	−1.259	1.189
GDP (log-diff)	250	0.011	0.023	−0.114	0.057
Density pharmacists (log-diff)	250	0.013	0.023	−0.053	0.135
Density MRI units (log-diff)	250	0.083	0.084	−0.092	0.445
Density GPs (log-diff)	250	0.012	0.025	−0.065	0.139
Density physicians (log-diff)	250	0.016	0.012	−0.024	0.075
Density specialists (log-diff)	250	0.023	0.017	−0.043	0.089
Density (acute) care beds (log-diff)	250	−0.019	0.015	−0.084	0.023
Death rate malignant neoplasms (log-diff)	250	−0.011	0.013	−0.047	0.031
Life expectancy (log-diff)	250	0.003	0.003	−0.006	0.014
Prevalence of overweight in adults (log-diff)	250	0.010	0.003	0.003	0.024
SHI	250	0.600	0.491	0	1
Legislatively required HTA	250	0.356	0.480	0	1
Generic prescription	250	0.496	0.501	0	1
Generic substitution	250	0.444	0.498	0	1
Reference price system	250	0.548	0.499	0	1
Pharmaceutical budgets for physicians	250	0.268	0.444	0	1
Electronic prescription	250	0.312	0.464	0	1
Information on prescription behaviour	250	0.564	0.497	0	1
*Extended sample*					
Public pharmaceutical expenditure (log-diff)	300	0.038	0.072	−0.322	0.433
Private pharmaceutical expenditure (log-diff)	300	−0.010	0.125	−1.259	1.189
GDP (log-diff)	300	0.012	0.022	−0.114	0.057
Density pharmacists (log-diff)	300	0.013	0.023	−0.077	0.135
Density MRI units (log-diff)	300	0.082	0.084	−0.092	0.524
Density GPs (log-diff)	300	0.011	0.024	−0.065	0.139
Density physicians (log-diff)	300	0.014	0.012	−0.024	0.075
Density specialists (log-diff)	300	0.021	0.017	−0.043	0.089
Density (acute) care beds (log-diff)	300	−0.019	0.016	−0.104	0.032
Death rate malignant neoplasms (log-diff)	300	−0.011	0.012	−0.047	0.031
Life expectancy (log-diff)	300	0.003	0.003	−0.006	0.014
Prevalence of overweight in adults (log-diff)	300	0.010	0.003	0.003	0.024
SHI	300	0.500	0.501	0	1
Legislatively required HTA	300	0.297	0.458	0	1
Generic prescription	300	0.580	0.494	0	1
Generic substitution	300	0.537	0.499	0	1
Reference price system	300	0.457	0.499	0	1
Pharmaceutical budgets for physicians	300	0.223	0.417	0	1
Electronic prescription	300	0.283	0.451	0	1
Information on prescription behaviour	300	0.470	0.500	0	1

*SHI* Social health insurance, *HTA* health technology assessment

For the construction of dummy variables indicating policy change with respect to each cost control measure category, diverse sources on the historical development of healthcare systems were used, among which the PPRI reports (DeSwaef & Antonissen, 2008; Peura et al., 2007; Redman & Köping Höggärd, 2007; Palnoch et al., 2007; Stargardt et al., 2008; Thomsen et al., 2008; Vogler et al., 2008), OECD reports (Belloni et al., 2016; Colombo et al., 2006; Moïse & Docteur, 2007) and Health Systems in Transition reports by the European Observatory (Busse & Riesberg, 2004; den Exter et al., 2004; Sandier et al., 2004; Glenngård et al., 2005; Corsens, 2007; Vuorenkoski, 2008; Marchildon, 2013) were the most valuable sources. In addition, experts from the individual countries were contacted for confirmation and clarification.

## Empirical strategy

Different expenditure control measures were implemented in different OECD member countries at different times. This allows us to make use of these differences across time and countries to isolate the associations of six types of expenditure control measures (Table 1) with PPE growth from confounding factors in the panel data setting. However, it is important to note that this study design does not allow for a causal interpretation of the estimated coefficients. The nature of the data adds another dimension to the problems of the statistical analysis of this issue: first, an abundance of potential covariates may lead to overfitting of the statistical models. Second, due to incomplete country time series and the resulting sample selection, spatial methods cannot be used to deal with the problem of contemporaneous cross-sectional correlation. We, therefore, propose a study design that allows addressing both issues.

### Error-term correction

Levin-Lin-Chu tests (Levin et al., 2002) indicate a unit root in the level time series of the dependent variable.^3^ The continuous variables are therefore transformed in log-differences. Our statistical framework starts from the time-series cross-sectional model:

1 $$\begin{aligned} y_{it} = X'_{it} \beta _{1} + D'_{it} \beta _{2} + \varepsilon _{it} \end{aligned}$$

with $i = 1,\ldots , N$ (countries), $t = 1,\ldots , T$ (years). The variable $y_{it}$ is PPE in log-difference and $X'_{it}$ is the set of log-differenced regressors given in Table 2, including a common intercept, and $D'_{it}$ is the set of dummy variables. The composite error term ${\varepsilon } \sim N(0,\sigma ^{2}\varOmega )$, where $\varOmega$ is a $NT \times NT$ positive definite matrix, allows for group-wise heteroscedasticity, common first-order serial correlation and time-invariant cross-sectional correlation^4^:

2 $$\begin{aligned} \varepsilon _{it} = \rho \varepsilon _{i,t-1} + u_{it} \end{aligned}$$

where $|\rho |<1$ is the common autocorrelation parameter and the zero-mean innovations $u_{it}$ are temporally independent and identically distributed.

The error covariance matrix of a balanced panel is given by

3 $$\begin{aligned} \varOmega = E \left[ \varepsilon \varepsilon ' \right] = \varSigma \otimes \varPi \end{aligned}$$

with

4 $$\begin{aligned} \varSigma = \begin{bmatrix} \sigma _{\varepsilon , 11} &{} \sigma _{\varepsilon , 12} &{} \cdots &{} \sigma _{\varepsilon , 1N} \\ \sigma _{\varepsilon , 21} &{} \sigma _{\varepsilon , 22} &{} \cdots &{} \sigma _{\varepsilon , 2N} \\ \vdots &{} \vdots &{} \ddots &{} \vdots \\ \sigma _{\varepsilon , N1} &{} \sigma _{\varepsilon , N2} &{} \cdots &{} \sigma _{\varepsilon , NN} \end{bmatrix} \end{aligned}$$

and

5 $$\begin{aligned} \varPi = \frac{1}{1-\rho ^2} \begin{bmatrix} 1 &{} \rho &{} \rho ^{2} &{} \cdots &{} \rho ^{T-1} \\ \rho &{} 1 &{} \rho &{} \cdots &{} \rho ^{T-2} \\ \rho ^{2} &{} \rho &{} 1 &{} \cdots &{} \rho ^{T-3} \\ \vdots &{} \vdots &{} \vdots &{} \ddots &{} \vdots \\ \rho ^{T-1} &{} \rho ^{T-2} &{} \rho ^{T-3} &{} \cdots &{} 1 \end{bmatrix} \end{aligned}$$

where $\varSigma$ is the $N \times N$ panel-by-panel covariance matrix and $\varPi$ is the $T \times T$ autocorrelation matrix.

Following Magnus & De Luca (2016) and Magnus et al. (2011), we correct for the nonspherical disturbances by pre-multiplying equation (1) by an estimate of $\varOmega ^{-\frac{1}{2}}$ under the normalization constraint $trace(\varOmega )=n$ based on ${\hat{\varOmega }}={\hat{\varSigma }}\otimes {\hat{\varPi }}$:

6 $$\begin{aligned} {\tilde{y}} = \tilde{X'} \beta _{1} + D' \beta _{2} + {\tilde{\varepsilon }} \end{aligned}$$

with $\tilde{X'} = {\hat{\varOmega }}^{-\frac{1}{2}} X'$, and the independent and identically distributed errors ${\tilde{\varepsilon }} = {\hat{\varOmega }}^{-\frac{1}{2}} \varepsilon$, all in the stacked form of dimension *NT*. Note that we have excluded the dummy variables $D'$ from the transformation to conserve their binary character as the pre-multiplication would otherwise only complicate the interpretation of the coefficient. Moreover, in contrast to the continuous covariates, the autocorrelation and cross-sectional correlation does not affect the policy dummies variables, justifying their exclusion. The estimates ${\hat{\rho }}$ and ${\hat{\varSigma }}$ are extracted from an estimation of the full linear model (1) including all continuous and dummy covariates with the PCSE-estimator [Panel-Corrected Standard Errors, see Beck and Katz (1995)], which preserves the Prais–Winsten transformation for autocorrelation but uses a sandwich estimator to incorporate cross-sectional dependence when calculating standard errors. The PCSE-estimator is shown to be the superior estimator in the current setting when the primary concern is hypothesis testing (Moundigbaye et al., 2018).

### Weighted-average least squares estimation

As numerous variables are potential candidates for inclusion in the regression model, the issue of model choice is not straight-forward to resolve and can have nonnegligible effects on the statistical properties of the estimators and hence the estimated coefficients (Magnus & Durbin, 1999; Danilov & Magnus, 2004; Moral-Benito, 2015). In this analysis, we use the weighted-average least squares (WALS) approach introduced by Magnus et al. (2010) which combines Bayesian and frequentist estimators. We have chosen the WALS approach for this analysis as the combination of these features gives this model averaging estimator an edge over strictly Bayesian and strictly frequentist model averaging estimators: in contrast to strictly Bayesian approaches, theoretical considerations determine the choice of priors in WALS that relate to admissibility, bounded risk, robustness and near-optimality in terms of minimax regret (De Luca et al., 2018). In addition, WALS presents a more explicit and transparent treatment of ignorance in the choice of priors (Magnus et al., 2010). As WALS uses a semiorthogonal transformation of the regressors, the computational burden is greatly reduced compared to other Bayesian or frequentist alternatives. We use the implementation of WALS in STATA by De Luca & Magnus (2011) in combination with a data set containing the pre-multiplied time series as per Eq. (6). A key aspect of the model selection in WALS is the distinction between focus and auxiliary regressors, which was first introduced in Danilov & Magnus (2004). The inclusion of focus regressors in the model is fixed based on theoretical considerations, while the inclusion of auxiliary regressors is mutable. Model uncertainty within this framework arises since different subsets of variables could be excluded from the model, leading to a trade-off between bias and precision in the estimators of the focus regressors. Thus, model selection takes place over the subset of auxiliary regressors, $k_{A}$, resulting in the model space ${\mathcal {M}}:= \{ {\mathcal {M}}_{j}, j= 1,\ldots ,2^{k_{A}} \}$ with $2^{k_{A}}$ possible models. Rewriting equation (6), the model ${\mathcal {M}}_{j}$ can be expressed as:

7 $$\begin{aligned} {\tilde{y}}= {\tilde{F}}'\beta _{F} + {\tilde{A}}'_{j} \beta _{Aj} + {\tilde{\varepsilon }}_{j} \end{aligned}$$

where ${\tilde{F}}_{j}$ is the matrix of the $k_{F}$ (transformed) focus regressors, ${\tilde{A}}_{j}$ is the matrix of the $k_{Aj}$ (transformed) auxiliary regressors, $\beta _{F}$ and $\beta _{Aj}$ are the corresponding parameters, and ${\tilde{\varepsilon }}_{j}$ is the vector of independent and identically distributed errors.

We fix the variables GDP, private pharmaceutical expenditure and the dummy for SHI systems as focus regressors. The remaining variables are considered auxiliary regressors resulting in a model space of 524,288 models, or 1125 billion models when including the country- and year-dummies. The policy variables were intentionally not chosen as focus regressors in order to avoid overfitting and ensure parsimony of the resulting model, although these are the main variables of interest.

In the key steps of the WALS estimation, the orthogonal $k_{A} \times k_{A}$ matrix *P* and a diagonal $k_{A} \times k_{A}$ matrix $\varLambda$ are computed such that $P'{\tilde{A}}'M_{{\tilde{F}}}{\tilde{A}}P=\varLambda$, where $M_{{\tilde{F}}}=I_{1}-{\tilde{F}}({\tilde{F}}'{\tilde{F}})^{-1}{\tilde{F}}'$ is a symmetric and idempotent matrix (Magnus et al., 2010). Using these matrices $Z_{{\tilde{A}}}={\tilde{A}}P\varLambda ^{-\frac{1}{2}}$ and $\gamma _{{\tilde{A}}}=\varLambda ^{\frac{1}{2}}P'\beta _{{\tilde{A}}}$ are defined such that $Z'_{{\tilde{A}}}M_{{\tilde{F}}}Z_{{\tilde{A}}}=I_{k_{A}}$ and $Z_{{\tilde{A}}}\gamma _{{\tilde{A}}}={\tilde{A}}\beta _{{\tilde{A}}}$. Applying the orthogonal transformation to the basic linear regression model (7) and using a Laplace estimator ${\hat{\eta }}_{j}$ for the theoretical t-ratio $\eta _{j} = \frac{\gamma _{{\tilde{A}}j}}{\sigma _{\varepsilon }}$ based on the Laplace prior distribution

8 $$\begin{aligned} \pi (\eta _{j};c)= \frac{c}{2} e^{-c|\eta _{j}|} \end{aligned}$$

with $c=log2$ such that the prior median of $\eta _{j}$ is zero and the prior median of $\eta _{j}^{2}$ is one (which reflects the notion of ignorance in the choice of priors), the resulting WALS estimators of the regression parameters $\beta _{{\tilde{F}}}$ and $\beta _{{\tilde{A}}}$ are given by

9 $$\begin{aligned}&{\tilde{\beta }}_{{\tilde{F}}} = ({\tilde{F}}'{\tilde{F}})^{-1}{\tilde{F}}'({\tilde{y}}-{\tilde{A}}{\tilde{\beta }}_{{\tilde{A}}}) \nonumber \\&{\tilde{\beta }}_{{\tilde{A}}} = s P \varLambda ^{-\frac{1}{2}} {\hat{\eta }} \end{aligned}$$

where *s* is derived from the estimator $s_{j}^{2}$ for $\sigma _{{\tilde{\varepsilon }}}^{2}$ in model ${\mathcal {M}}_{j}$.

## Results

Table 3 provides a comparison of the *t-ratios* of WALS regressions for the two samples with and without country- and year-dummies and Table 4 presents the respective coefficient estimates. Columns (1) and (3) present the results for the core sample of European countries and columns (2) and (4) for the extended sample including Canada and the US. Note that as the WALS estimation is a model averaging technique and therefore, as Magnus and De Luca (2016, p. 118)note, “*does not select a single model out of the available set of models*”, but rather allows each model to contribute information on the parameters of interest. Accordingly, we present the estimated coefficients of all potential control variables in Tables 3 and 4.

**Table 3:** Comparison *t*-ratios for the core (Europe) and extended sample (Europe $+$ Canada and USA)

Sample	Europe	Europe $+$ Canada and USA	Europe	Europe + Canada and USA
Dependent variable				
Public pharmaceutical expenditure	*t*-ratio	*t*-ratio	t-ratio	*t*-ratio
	(1)	(2)	(3)	(4)
Private pharmaceutical expenditure^a^	−11.20	−11.00	−11.15	−10.70
Constant^a^	1.43	1.46	1.69	0.98
GDP^a^	4.37	2.53	3.65	2.86
Electronic prescription system	−2.41	−2.61	−0.13	0.00
SHI^a^	−0.91	−1.50	−0.95	−0.25
Density MRI units	1.67	1.82	0.92	1.54
Pharmaceutical budget for physicians	−1.00	−0.83	−1.96	−1.29
Generic substitution (lag)	−1.58	−1.70	−0.71	−1.04
Density specialists	−0.08	−0.81	0.14	−0.36
Density generalist medical practitioners	−1.27	−1.17	−1.59	−1.96
Density physicians	0.81	1.12	0.75	1.24
Information on prescription behaviour	−1.00	−1.53	−0.58	−0.88
Death rate malignant neoplasms	0.54	0.45	0.30	0.26
Density pharmacists	0.67	0.98	1.01	1.07
Density (acute) care beds	−0.10	−0.88	0.30	−0.29
Reference price system (lag)	−0.60	−0.48	−0.02	−0.23
Generic prescription	−0.31	0.04	−0.45	0.08
Generic substitution	1.20	1.09	0.95	0.91
Reference price system	0.63	0.64	0.31	0.33
Life expectancy	−0.61	−0.06	−0.60	0.30
Generic prescription (lag)	0.20	0.09	0.67	0.83
Prevalence of overweight in adults	1.83	1.90	1.01	0.38
Legislatively required HTA	0.11	0.08	−0.84	−0.88
*N*	240	288	240	288
Country- and time-fixed effects	No	No	Yes	Yes
Model space	524,288 models		1125 billion models^b^	

Note that the sample size size is reduced by 10 and 12 observations respectively, due to the inclusion of variables with a one-year lag

^a^Fixed inclusion

^b^Country-dummies for CAN, UK and USA omitted because of collinearity

**Table 4:** Results of the WALS regression for the core (Europe) and the extended sample (Europe + Canada and USA)

Results statistical analysis				
Sample	Europe	Europe $+$ Canada and USA	Europe	Europe + Canada and USA
Dependent variable				
Public pharmaceutical expenditure	(1)	(2)	(3)	(4)
GDP	0.865$$***$$	0.509$$**$$	0.953$$***$$	0.794$$***$$
	[0.475, 1.254]	[0.112, 0.906]	[0.438, 1.468]	[0.246, 1.342]
Private pharmaceutical expenditure	−0.313$$***$$	−0.295$$***$$	−0.303$$***$$	−0.286$$***$$
	[−0.368, −0.258]	[−0.348, −0.243]	[−0.356, −0.249]	[−0.338, −0.233]
SHI	−0.00927	−0.0164	−0.0366	−0.00978
	[−0.0293, 0.0107]	[−0.0380, 0.00516]	[−0.113, 0.0396]	[−0.0860, 0.0665]
Legislatively required HT	0.00105	0.000818	−0.0131	−0.0140
	[−0.0169, 0.0190]	[−0.0182, 0.0198]	[−0.0439, 0.0177]	[−0.0454, 0.0174]
Density pharmacists	0.0690	0.105	0.111	0.122
	[−0.135, 0.272]	[−0.107, 0.318]	[−0.106, 0.328]	[−0.103, 0.348]
Density MRI units	0.0637$^*$	0.0719$^*$	0.0371	0.0739
	[−0.0117, 0.139]	[−0.00611, 0.150]	[−0.0423, 0.117]	[−0.0206, 0.168]
Death rate malignant neoplasms	0.103	0.0919	0.0599	0.0567
	[−0.275, 0.481]	[−0.307, 0.490]	[−0.336, 0.456]	[−0.365, 0.479]
Density physicians	0.218	0.311	0.202	0.376
	[−0.313, 0.749]	[−0.235, 0.856]	[−0.327, 0.732]	[−0.222, 0.974]
Density GPs	−0.163	−0.154	−0.196	−0.254*
	[−0.415, 0.0894]	[−0.413, 0.105]	[−0.440, 0.0471]	[−0.509, 0.00107]
Density specialists	−0.0154	−0.153	0.0236	−0.0726
	[−0.375, 0.344]	[−0.523, 0.218]	[−0.317, 0.364]	[−0.474, 0.329]
Density (acute) care beds	−0.0201	−0.184	0.163	−0.0657
	[−0.404, 0.364]	[−0.594, 0.226]	[−0.229, 0.555]	[−0.518, 0.386]
Life expectancy	−0.569	−0.0738	−0.789	0.477
	[−2.396, 1.258]	[−2.557, 2.410]	[−3.381, 1.803]	[−2.659, 3.612]
Prevalence of overweight in adults	1.801$*$	1.573$*$	1.532	0.581
	[−0.138, 3.740]	[−0.0554, 3.201]	[−1.447, 4.511]	[−2.469, 3.631]
Electronic prescription	−0.0245$$**$$	−0.0287$$***$$	−0.00181	−0.0000362
	[−0.0445, −0.00447]	[−0.0505, −0.00700]	[−0.0288, 0.0252]	[−0.0291, 0.0290]
Information on prescription behaviour	−0.00994	−0.0166	−0.00778	−0.0123
	[−0.0296, 0.00970]	[−0.0379, 0.00475]	[−0.0343, 0.0188]	[−0.0402, 0.0155]
Pharmaceutical budgets for physicians	−0.00915	−0.00799	−0.0578$*$	−0.0388
	[−0.0272, 0.00887]	[−0.0270, 0.0111]	[−0.116, 0.000391]	[−0.0980, 0.0205]
Generic prescription	−0.00772	0.00106	−0.0115	0.00209
	[−0.0574, 0.0420]	[−0.0502, 0.0523]	[−0.0614, 0.0384]	[−0.0507, 0.0549]
Generic substitution	0.0279	0.0264	0.0228	0.0235
	[−0.0181, 0.0739]	[−0.0213, 0.0741]	[−0.0246, 0.0702]	[−0.0272, 0.0743]
Reference price system	0.0129	0.0140	0.00606	0.00680
	[−0.0274, 0.0533]	[−0.0290, 0.0570]	[−0.0328, 0.0449]	[−0.0344, 0.0480]
Generic prescription (lag)	0.00500	0.00238	0.0170	0.0224
	[−0.0454, 0.0554]	[−0.0498, 0.0545]	[−0.0328, 0.0668]	[−0.0308, 0.0756]
Generic substitution (lag)	−0.0371	−0.0417$*$	−0.0162	−0.0257
	[−0.0836, 0.00931]	[−0.0899, 0.00657]	[−0.0612, 0.0289]	[−0.0742, 0.0229]
Reference price system (lag)	−0.0121	−0.0105	−0.000388	−0.00493
	[−0.0518, 0.0277]	[−0.0532, 0.0322]	[−0.0394, 0.0387]	[−0.0465, 0.0367]
Constant	0.0191	0.0222	0.0691$*$	0.0418
	[−0.00717, 0.0453]	[−0.00787, 0.0523]	[−0.0117, 0.150]	[−0.0426, 0.126]
*N*	240	288	240	288
Country- and time-fixed effects	No	No	Yes	Yes

Note that the sample size size is reduced by 10 and 12 observations respectively, due to the inclusion of variables with a one-year lag. 95%-Confidence intervals in parentheses

*$$p <0.1$$, **$$p <0.05$$

***$$p<0.01 $$

The results suggest that the effects most demand-side policy measures are not as clear once compared across countries. Electronic prescription has the highest absolute t-ratios of the policy measures under investigation when not including country- and year-dummies. It shows the strongest consistent estimated coefficient in the WALS regressions with around 2.5% lower annual growth depending on the sample, though both size and significance vanish when including country- and year-dummies.

The estimated coefficient for pharmaceutical budgets and generic substitution (lagged by one year are of similar size (roughly 5% lower annual growth), but in contrast to electronic prescription are only significant at the 10%-level of confidence in the extended sample without country- and time-fixed effects (pharmaceutical budgets) and the core sample with country- and time-fixed effects (lagged generic substitution), respectively. Information systems on prescription behaviour have relatively high t-ratios in the core sample, but also fail to reach significance and the coefficient is somewhat smaller. It is a noteworthy result that reference price systems and generic prescription have only relatively low t-ratios and hence no statistically significant coefficients.

We further find a strong and highly significant association between a higher cost-sharing and PPE growth, suggesting that a 1% increase in private pharmaceutical expenditure growth reduces PPE growth by roughly 0.3%. It is of course by itself not a surprising finding that a higher share of private expenditure will ultimately lower the share of public expenditure. However, the size and significance of the coefficient are robust to both the inclusion of non-European countries in the sample as well as the inclusion of country- and year-dummies. Among the control variables, we find a positive association between GDP and PPE growth. The prevalence of overweight in the adult population as a proxy for chronic disease burden, and the density of MRI units as a proxy for technological progress have a minor positive association with PPE growth as well, though only when not including country- and year-dummies. In contrast, a higher density of generalist medical practitioners is associated with lower PPE growth, though the association is only significant in the extended sample when country- and year-dummies are included. For the remaining control variables we do not find statistically significant effects on PPE growth.

## Discussion

Rising pharmaceutical expenditure puts a strain on publicly financed healthcare systems, an issue that is expected to again gain relevance in the future after a period of relative calm, not least due to novel therapeutic approaches gradually becoming available (Belloni et al., 2016). We analysed policies enacted in different countries over the course of 25 years in terms of their effectiveness to curb PPE growth for retail pharmaceuticals. Our empirical analysis suggests that reductions in retail PPE growth are achievable, both by patient-level cost-sharing schemes and demand-side control measures. But not all policy measures seem to have been equally successful, and what has worked in one country might not work as well in a different setting.

While some of our findings are well in line with the literature like the positive link between GDP and PPE growth (Shaikh & Gandjour, 2019; Clemente et al., 2008; Okunade & Suraratdecha, 2006) or the negative link between patient cost-sharing and PPE growth, our results with respect to demand-side expenditure control measures are more unexpected. Only two physician-level behaviour expenditure control measures, electronic prescription and pharmaceutical budgets, have a statistically significant negative association with PPE growth, though the association depends both on the inclusion of country- and year-dummies in the statistical model as well as the sample choice. For information systems on prescription behaviour, we do not find an association with PPE growth in our analysis.

Among system-level substitution measures aiming at promoting the use of generics (i.e. generic substitution and generic prescription), only generic substitution (with a one-year lag) has notable t-ratios and has a significant association with PPE growth in the extended sample when including country- and year-dummies. For system-level price-control measures in the form of reference price systems, no significant association with PPE growth is identified. The latter is a particularly interesting finding, as reference price systems are widely used in OECD countries and studies like Acosta et al. (2014) suggest that reference price systems can lead to a reduction of pharmaceutical expenditure in the short term by shifting use from cost-share drugs to reference drugs, although the authors themselves point to the low quality of the evidence. Our results hence suggest that the effectiveness of reforms depends on the timing and the context of their implementation, both of which is not adequately captured in meta-studies.

Our results highlight that with a relatively large impact, patient cost-sharing has been an effective policy tool to curb the growth of PPE, though it comes with a certain risk for social equity and increasing costs in other sectors down the line. Over the last years, several countries have increased patient cost-sharing for retail pharmaceuticals, including France and Sweden (Belloni et al., 2016). Patient cost-sharing affects PPE in several ways. First, an obvious effect is that, ceteris paribus, higher cost-sharing increases the share of private expenditure in total expenditure on pharmaceuticals shifting the balance between public and private expenditure. Second, cost-sharing can also influence patients’ consumption of pharmaceuticals, i.e higher cost-sharing could reduce patients’ use of pharmaceuticals. There are two sides to this coin: while in some instances, decreased use of pharmaceuticals can be desirable (for instance, if patients do not ask for antibiotics in cases where use might not be appropriate), adverse effects can occur if adherence to treatment plans is lowered when drugs are less affordable due to higher cost-sharing (Austvoll-Dahlgren et al., 2008). The cost savings for PPE may hence be offset by increased costs of protracted treatment in other health-related budgets, e.g. when more expensive acute care is need that could have otherwise been avoided, as well as in other sectors, e.g. productivity losses through presenteeism or absenteeism. These adverse effects could be counterbalanced as patients might be more inclined to opt for lower-priced pharmaceuticals, i.e. generics, when the level of cost-sharing is high (Belloni et al., 2016). As empirical evidence from Korea suggests, increased cost-sharing can contribute to lowering per patient expenditure on pharmaceuticals without notably affecting utilisation cost-sharing system within a country next to the availability of generic alternatives. It is not an unreasonable conjecture that lump-sum payments per package do not cause such effects. Patient cost-sharing schemes nevertheless bear a high risk of adverse societal effects as the financial burden imposed on patients can be substantial, especially in lower-income countries (Vogler et al., 2019). Hence, patient cost-sharing is far from being a "one-size-fits-all" solution. Accordingly, a wide spectrum of different cost-sharing schemes exists across OECD countries (Barnieh et al., 2014; World Health Organization, 2018).

We want to briefly discuss some limitations pertaining to data and study design that we consider important for the interpretation of our results. By aggregating cost control policies into the six principal categories, some informational content is necessarily lost. An example would be that reference price systems differ in details between countries. In our study design, we make a trade-off between informational content and usability of the policy variables to ensure that a meaningful interpretation of the results is possible. Insufficient aggregation limits the power of the statistical inference as the number of observations is reduced. In case policy variables coincide with country variables, the estimated significant policy effects could be masked by unobserved country-specific characteristics. Moreover, although our study design always provides a counter-factual for the different policy measures to be compared against, the estimated effects cannot be interpreted as causal. Several cost containment measures also had to be omitted from the analysis for practical reasons, for instance in case of pilot projects, or policies targeting only specific types of pharmaceuticals, or when the policy itself cannot be observed, like in the case of country-specific price differentials due to rebates. Espin et al. (2018) suggest that the latter in particular have a strong impact in the forecasting the growth of pharmaceutical expenditure. Indeed, confidential and complex discounts have become more widespread over recent years, but more often concern speciality drugs than primary care drugs (Morgan et al., 2017). We therefore expect a somehwat lower impact in the context of retail pharmaceuticals, which are the focus of our study.

Another important aspect of our study approach is that we are only able to include *explicitly formulated* policies in contrast to implicit cost-control measures. For instance, a country may not follow a formal generic substitution policy as defined in our study, but could yet otherwise incentivise the use of generics. To a certain extent, these effects should, however, be absorbed by the addition of country-specific dummies in the WALS regression. There may arguably exist additional synergies between cost control measures included in the study and those that could not be included. However, an exploration of these is beyond the scope of our study approach. Along these lines, a potential path for future research would be to account for the fact that there often is not one standard way of implementing a certain type of policy (e.g. indicative versus mandatory generic prescription) and to focus cross-country panel-analysis on specific categories of policies to highlight best-practice examples, which may also allow exploration of synergies between individual policy measures.

The sample selection through the requirement of sufficiently complete time series may also distort the estimates given the comparatively small number of countries under investigation. A clear identification of the impact of some policy variables is further limited by relying on a small number of country observations only. In view of the limitations concerning our study design, we stress that our results should be read as conflicting explorative empirical evidence rather than definite evidence in favor of discarding specific policy measures. A further limitation of our approach is the impossibility of distinguishing between PPE reductions due to desired or undesired effects, for instance when the level of patient cost-sharing is increased. Last but not least, we want to stress again that the data used in our analysis do not cover medication dispensed in hospitals, therefore only policies affecting retail pharmaceutical expenditure outside hospitals are analysed and results must be interpreted accordingly. Overall, improving data availability would greatly strengthen the evidence base for policy making.

Policy makers must proceed with great care when implementing patient-level cost sharing schemes to ensure social equity. The importance of this aspect is vividly underlined by recent research of the WHO on unmet need and financial hardship in European healthcare systems (Thomson et al., 2019). To maximise impact while minimising adverse effects, reforms must be tailor-made to current circumstances and future developments within a country. That is, whether the level of patient cost-sharing is already high in a country to begin with, or if measures are in place to protect lower-income patients from adverse effects. Against this background, the introduction of demand-side measures targeting behaviour at the physician-level, or prices at the healthcare system-level, or fostering substitution of brand drugs with generics appears a safer choice regarding economic and social equity, even though their expected impact is likely more limited and may not be free of accompanying adverse effects either.

## A Appendix

See Appendix Fig. 2.

## Footnotes

1 Strictly speaking, this argument is valid also for generic prescription and generic substitution as for innovative drugs no generic versions are available.

2 The sources for this variable are the same as for the main policy variables described below. Additional information was taken from the HTA country profiles published by the World Health Organization.

3 The null hypothesis of unit roots in the panel is not rejected without and with trend: *p*-value = 0.2656 and 0.3066, respectively. The Levin–Lin–Chu unit-root tests with and without trend reject the null hypothesis for all log-differenced time series, thus excluding I(2)-processes.

4 A Breusch-Pagan test (Breusch & Pagan, 1979) for heteroscedasticity across panels rejects the null hypothesis of constant variance in the data in levels, but not in log-differences. The single log-differenced time series were tested for serial correlation using the bias-corrected *Q*(*P*)-statistics as proposed in Born and Breitung (Born & Breitung, 2016). The results suggest the presence of first-order autocorrelation in all but three time series (private pharmaceutical expenditure, and pharmacists and specialists per 1000 population). The test is preferred to the Wooldridge test for autocorrelation in panel data (Wooldridge, 2002; Drukker, 2003), as it has more power in panels with small *N*. We model a common AR(1)-process of the error term as advised by Beck and Katz (1995) for models with moderate T. A Pesaran test of cross-sectional independence (Pesaran, 2004) strongly suggests the presence of time-invariant cross-sectional correlation in the log-differenced data.
